# Cognitive Symptoms and Overall Functioning During Major Depressive Episodes: Correlation Analysis of Patients With Unipolar and Bipolar Disorders

**DOI:** 10.1155/da/7231049

**Published:** 2026-05-14

**Authors:** Giorgia Porceddu, Camilla Garrone, Giuseppe Maina, Gianluca Rosso

**Affiliations:** ^1^ Department of Neurosciences, San Luigi Gonzaga University Hospital, Orbassano, Turin, 10043, Italy; ^2^ Department of Neurosciences, University of Turin, Turin, 10126, Italy, unito.it

## Abstract

**Aim:**

This cross‐sectional observational study was designed to characterize global functioning in patients with unipolar depression (UD) and bipolar depression (BD), focusing on the relationship between functional impairment and objectively assessed and subjectively perceived cognitive deficits, as well as to potential domain‐specific cognitive–functional patterns across diagnostic groups.

**Methods:**

Individuals experiencing a major depressive episode (MDE) in the context of major depressive or bipolar disorder were recruited. Global functioning was assessed with the Functional Assessment Short Test (FAST), objective cognition with the Screen for Cognitive Impairment in Psychiatry (SCIP), and subjective cognition with the Perceived Deficits Questionnaire–Depression–5‐item (PDQ‐D‐5). Group differences were analyzed using *χ* ^2^ tests and ANCOVA, adjusting for illness duration and age at onset. Associations between cognitive measures and global functioning were examined using Pearson’s correlations.

**Results:**

A total of 102 patients were recruited: 54 with UD, 48 with BD. Clinically relevant functional impairment was observed in 87% of UD patients and 93.7% of BD patients. BD individuals showed greater global functional impairment than UD patients, with higher FAST total scores (44.5 ± 23.1 vs. 33.5 ± 14.6, *p* = 0.006) and worse functioning across most domains. Global functioning was strongly associated with subjectively perceived cognitive difficulties in both UD (*ρ* = 0.609, *p* < 0.001) and BD (*ρ* = 0.475, *p* < 0.001), whereas no significant associations were found with objective cognitive performance. Domain‐specific analyses revealed different patterns of association, with attention and organization related to functioning in UD and retrospective memory in BD.

**Conclusion:**

MDEs in unipolar and bipolar disorders are associated with marked functional impairment. Perceived cognitive difficulties may impact daily functioning more than objectively assessed deficits, highlighting their clinical relevance. The distinct cognitive–functional profiles in UD and BD patients underscore the importance of domain‐specific assessments to guide interventions targeting both symptom remission and functional recovery.

## 1. Introduction

Major depressive episodes (MDEs) represent the phase during which individuals with mood disorders spend the greatest proportion of time in an active state of illness, thereby exerting a substantial influence on the overall course of the disorder [1, 2]. MDEs impose a profound and pervasive burden, with a marked impact on global functioning [3–5]. The prevalence of functional impairment during MDEs is high, with evidence reporting that up to 75% of individuals with bipolar disorder or unipolar major depressive disorder (MDD) experience significant social and occupational dysfunction [6]. Notably, global functioning frequently remains impaired despite symptomatic remission, with persistent deficits observed during euthymic phases in both individuals with unipolar and bipolar disorders [7, 8].

With respect to the depressive phase and patients’ functional capacity, earlier research suggested that impairments in global functioning and quality of life were primarily driven by core depressive symptoms, such as anhedonia, psychomotor retardation, fatigue, and low motivation [9–12]. More recent research has increasingly focused on the impact of cognitive deficits on functional outcomes, consistently demonstrating a close relationship between cognitive impairment and global functioning. This association has been reported in both unipolar depression (UD) and bipolar depression (BD); however, its magnitude appears to be influenced by several confounding factors, including illness severity and chronicity [13, 14]. Furthermore, accumulating evidence indicates a differential contribution of objective and subjective cognitive measures to functional outcomes. Specifically, subjective cognitive deficits showed a consistent association with poorer global functioning, whereas objectively assessed cognitive impairments did not appear to exhibit a comparable relationship [15, 16].

Despite this growing recognition, findings on the relationship between cognitive dysfunction and global functioning during MDEs remain heterogeneous and, at times, contradictory. Notably, existing studies are frequently limited by an exclusive focus on either UD or BD, the inclusion of mixed samples comprising both depressed and euthymic individuals, or the assessment of either objective or subjective cognitive functioning alone, without integrating both dimensions within the same sample [15–18]. The limited evidence is further affected by substantial heterogeneity in the neurocognitive assessment methods used to assess objective cognitive performance. Studies have employed a wide range of instruments that differ in scope and sensitivity, with many assessing only selected cognitive domains or relying on measures originally developed for other clinical populations [13, 17].

Methodological differences in the assessment of cognitive and functional deficits, together with the lack of direct comparisons between unipolar and bipolar populations, contribute to confounding and occasionally contradictory findings, thereby complicating our understanding of the impact of cognitive deficits on overall functioning across diagnostic groups.

Within this broader context, the present study is designed as a descriptive and exploratory investigation aiming to:

1. Characterize overall functioning in patients with UD versus BD, hypothesizing lower functioning in BD patients.
2. Describe the relationship between global functioning and objectively assessed vs. subjectively perceived cognitive impairment, expecting both to be associated with functioning.
3. Explore whether specific cognitive domains impact on functioning.

## 2. Materials and Methods

### 2.1. Study Design and Patients

This study employed a cross‐sectional observational design to investigate individuals experiencing an MDE within the course of MDD or bipolar disorder. Eligible participants were between 18 and 65 years of age, had completed at least 8 years of formal education, and met DSM‐5‐TR criteria for a primary MDE within a longitudinal diagnostic framework of MDD or BD. Recruitment was performed consecutively among all patients with UD or BD referred to the Psychiatric Unit of San Luigi Gonzaga University Hospital (University of Turin, Italy) between December 2024 and August 2025. Specifically, participants were recruited from the acute psychiatric inpatient ward and specialized outpatient clinics for mood disorders, capturing a real‐world spectrum of illness severity and enhancing external validity. To ensure diagnostic precision, all participants were evaluated by experienced psychiatrists using DSM‐5‐TR criteria within a longitudinal diagnostic framework, which included the integration of medical records and a semi‐structured interview commonly utilized in clinical practice and previous research [19, 20]. Only patients with a confirmed primary diagnosis of MDD or BD were included. Individuals with uncertain diagnoses, a history of organic brain disorders or significant head trauma, intellectual disability, alcohol or substance use disorders, or dementia and other neurodegenerative diseases were excluded. No refusals occurred during the recruitment period. For the final sample, complete sociodemographic, clinical, and psychometric data were collected systematically, with no missing values for any variables included in the analyses. Written informed consent was obtained from all participants following comprehensive explanation of the study aims and procedures. The study was approved by the local Ethics Committee of the University Hospital San Luigi Gonzaga of Orbassano, Turin, Italy.

### 2.2. Assessments and Procedures

Sociodemographic and clinical data at study entry were systematically gathered for all participants. Clinical profiles were reconstructed using data extracted from medical records and further enriched through a semi‐structured interview designed by the research team, which has been routinely implemented in both clinical practice and previous studies [19, 21, 22].

Assessment of depressive symptoms and cognitive functioning was conducted through a combination of subjective and objective instruments. Self‐report measures comprised the Perceived Deficits Questionnaire–Depression–5‐item (PDQ‐D‐5), administered to evaluate perceived cognitive difficulties, and the Beck Depression Inventory–Second Edition (BDI‐II) [23, 24], employed to quantify the severity of depressive symptomatology. The PDQ‐D‐5, originally developed for multiple sclerosis and subsequently validated in mood disorders [25, 26], captures four dimensions of cognitive functioning: attention and concentration, retrospective memory, prospective memory, and planning/organization. In the present investigation, the Italian short form (5‐item version) was adopted, providing a rapid yet reliable tool for the assessment of subjective cognitive impairment [27].

Objective assessments of cognitive and depressive dimensions were carried out using standardized clinician‐administered instruments. Cognitive functioning was measured with the Screen for Cognitive Impairment in Psychiatry (SCIP), whereas depressive symptom severity was evaluated with the Hamilton [28] Depression Rating Scale (HAM‐D). The SCIP is a brief pencil‐and‐paper battery specifically developed to provide a rapid and objective quantification of cognitive deficits in both affective and psychotic disorders [29, 30]. In the present study, the Italian version was employed, encompassing five domains frequently impaired in psychiatric populations: immediate verbal learning (VLT‐I), working memory (WMT), verbal fluency (VFT), delayed verbal learning (VLT‐D), and processing speed (PST) [31].

Global functioning was assessed with the Functional Assessment Short Test (FAST), a validated instrument extensively applied in psychiatric research and clinical practice, particularly in populations with affective disorders. The scale can be administered either as a clinician‐rated or self‐report measure and is specifically designed to assess impairments in everyday functional abilities [32, 33]. In the present study, the scale was administered in its clinician‐rated format, thereby ensuring that functional outcomes were evaluated independently of patients’ subjective perceptions and strengthening the interpretability of their relationship with objective and self‐reported cognitive difficulties. It comprises 24 items organized into six domains: autonomy, occupational functioning, cognitive functioning, financial management, interpersonal relationships, and leisure activities. Higher scores reflect greater levels of functional impairment, providing a quantitative overview of global dysfunction. According to validated thresholds, total scores below 15 denote preserved global functioning, whereas values equal to or above 15 indicate impairment [34].

### 2.3. Statistical Analysis

Continuous variables describing sociodemographic and clinical characteristics were reported as mean values with standard deviations (SDs), whereas categorical variables were expressed as absolute counts and relative percentages. Based on primary diagnosis, participants were classified into two distinct groups: those with UD and BD.

Group differences were examined using *χ* ^2^ tests for categorical variables, while one‐way ANCOVA was employed for continuous measures (while controlling for duration and age at onset of illness). Associations between subjective and objective cognitive performance—both in terms of global indices and specific cognitive subdomains—and measures of global functioning were examined using Pearson’s correlation coefficients.

A two‐tailed *p*‐value < 0.050 was considered statistically significant for all comparisons. Analyses were performed using the Statistical Package for the Social Sciences (SPSS, Version 28.0; IBM Analytics).

To further strengthen the robustness of the findings, a sensitivity analysis was undertaken using ANCOVA, with the number of prior depressive episodes and the total number of affective episodes included as additional covariates to account for the potential confounding effects of illness recurrence and chronicity.

## 3. Results

### 3.1. Clinical Characteristics of the Sample

**Figure 1:**
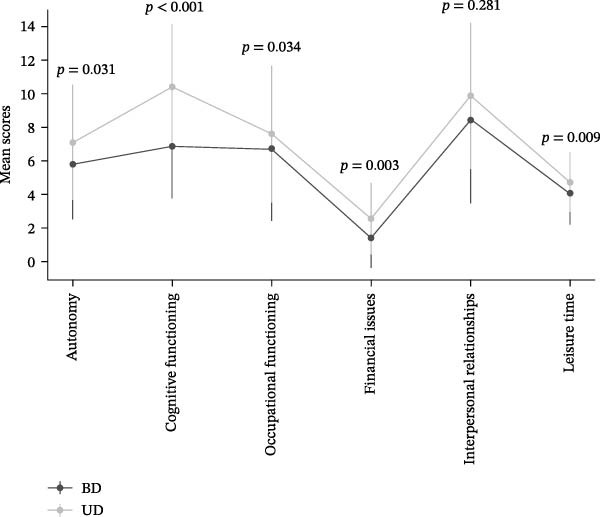
Differences in global functioning across FAST subdomains in UD and BD patients.

The study sample included 102 participants, consisting of 54 individuals with UD and 48 with BD. The BD and UD cohorts showed no meaningful differences across core sociodemographic characteristics—such as age, gender, and years of education—nor across lifestyle‐related risk factors, including smoking habits and physical inactivity. Regarding clinical characteristics, individuals with BD exhibited an earlier illness onset (28.3 ± 12.1 vs. 35.6 ± 15.5 years, *p* = 0.005) and a greater lifetime number of both total affective episodes (11.5 ± 10.6 vs. 3.2 ± 2.4, *p*  < 0.001) and MDEs (6.8 ± 5.0 vs. 3.2 ± 2.3, *p* = 0.002) compared with participants with UD. No significant differences were found between the two patient groups with respect to other mood disorder characteristics—including duration of untreated illness, prevalence of psychiatric family history, and medical and psychiatric comorbidities—as well as features of the current depressive episode—such as ongoing polypharmacy and treatment resistance. The duration of the current illness tended to be longer in patients with BD compared to those with UD. Although this difference did not reach formal statistical significance (*p* = 0.054), it may reflect the typically more prolonged depressive episodes observed in bipolar disorders. This is clinically relevant because depressive episodes in bipolar patients may be more difficult to detect early, given the overlap with previous hypomanic or mixed symptoms, which can delay diagnosis and treatment [35]. Furthermore, depressive symptom severity (HAMD‐D) and cognitive performance (SCIP) did not differ significantly between the two patient groups (see Tables 1 and 2); in contrast, global functioning (FAST) appeared more impaired in individuals with BD, both overall and across its major subdomains (see Figure 1 and Table 2). Additional information on the sociodemographic and clinical characteristics of the sample is presented in Tables 1 and 2.

**Table 1:** Sociodemographic and clinical characteristics of the sample.

Sociodemographic and clinical variables	UD (*n* = 54) mean ± SD or *n* (%)	BD (*n* = 48) mean ± SD or *n* (%)	*F*	*p*‐Value
Age	50.61 ± 16.221	52.23 ± 14.980	0.640	0.426
Sex (female)	40 (74.1)	35 (72.9)	0.017	0.895
Years of education	12.41 ± 3.248	13.52 ± 3.235	1.828	0.180
Married	25 (51.0)	21 (43.8)	2.768	0.429
Current employed	26 (50.0)	21 (50.0)	0.000	1.00
Smoker	11 (20.4)	16 (33.3)	2.194	0.139
Physical activity	11 (20.4)	10 (20.8)	0.003	0.954
Psychiatric disorder characterization				
Age at onset (years)	35.56 ± 15.51	28.30 ± 12.09	5.269	**0.005**
Number of total episodes	3.17 ± 2.42	11.53 ± 10.64	15.037	**<0.001**
Number of hypomanic episodes	__	2.19 ± 3.11	__	__
Number of manic episodes	__	0.66 ± 1.67	__	__
Number of depressive episodes	3.21 ± 2.35	6.77 ± 5.01	10.815	**0.002**
Duration of untreated illness (years)	6.6 ± 10.3	17.1 ± 19.6	2.238	0.138
Family history of psychiatric diseases	29 (53.7)	31 (64.6)	1.242	0.265
Medical comorbidity	41 (75.9)	40 (83.3)	0.853	0.356
Psychiatric comorbidity	18 (33.3)	15 (31.3)	0.050	0.822
Current major depressive episode				
Duration (days)	129.75 ± 229.85	77.81 ± 89.58	4.165	0.054
Treatment resistant depression	7 (14.0)	13 (28.3)	2.954	0.086
Objective cognitive impairment	41 (75.9)	33 (68.7)	0.657	0.418
Psychopharmacological treatment (current episode)				
Monotherapy	14 (25.9)	7 (14.6)	2.966	0.085
Polytherapy	40 (74.1)	41 (85.4)	2.966	0.085
HAM‐D score	17.96 ± 6.45	20.35 ± 9.35	2.616	0.109
HAM‐A score	12.40 ± 5.42	14.06 ± 6.62	0.920	0.340
YMRS score	2.80 ± 4.60	3.29 ± 3.03	0.148	0.702
CGI‐S score	4.35 ± 0.79	4.56 ± 1.05	3.078	0.083

*Note:* HAM‐A, hamilton anxiety rating scale; HAM‐D, hamilton depression rating scale. The bold values indicate statistically significant differences (*p* < 0.05).

Abbreviations: BD, bipolar depression; CGI‐S, clinical global impression–severity; SD, standard deviation; UD, unipolar depression; YMRS, young mania rating scale.

**Table 2:** Subjective and objective cognitive performance and global functioning.

Measures	UD (*n* = 54) mean (SD)	BD (*n* = 48) mean (SD)	*p*‐Value
PDQ‐D‐5 total score	8.59 ± 4.47	11.25 ± 3.37	**0.001**
Organization	1.93 ± 1.45	2.90 ± 1.13	**<0.001**
Attention	2.48 ± 1.51	3.42 ± 0.92	**<0.001**
Orientation	1.93 ± 1.52	2.27 ± 1.43	0.217
Retrospective memory	1.19 ± 1.36	1.42 ± 1.32	0.369
Prospective memory	1.07 ± 1.29	1.29 ± 1.25	0.645
SCIP total score	64.06 ± 13.82	65.04 ± 15.14	0.870
VLT‐I	18.04 ± 4.16	17.46 ± 4.67	0.141
WMT	19.07 ± 4.29	18.96 ± 3.84	0.863
VFT	14.93 ± 4.46	16.65 ± 5.44	0.249
VLT‐D	5.19 ± 2.29	4.31 ± 2.43	**0.038**
PST	7.43 ± 3.15	8.08 ± 3.45	0.201
FAST total score	33.54 ± 14.63	44.52 ± 23.13	**0.006**
Autonomy	5.81 ± 3.30	7.10 ± 3.43	**0.031**
Cognitive functioning	6.85 ± 3.07	10.40 ± 3.75	**<0.001**
Occupational functioning	6.72 ± 4.29	7.60 ± 4.07	**0.034**
Financial issues	1.41 ± 1.77	2.54 ± 2.12	**0.003**
Interpersonal relationships	8.44 ± 4.91	9.88 ± 4.39	0.281
Leisure time	4.07 ± 1.87	4.73 ± 1.78	**0.009**

*Note:* PDQ‐D‐5, perceived deficits questionnaire–depression–5‐items. The bold values indicate statistically significant differences (*p* < 0.05).

Abbreviations: BD, bipolar depression; FAST, functioning assessment short test; PST, psychomotor speed test; SCIP, screen for cognitive impairment in psychiatry; SD, standard deviation; UD, unipolar depression; VFT, verbal fluency test; VLT‐D, verbal learning test delayed; VLT‐I, verbal learning test immediate; WMT, working memory test.

### 3.2. Global Functioning in UD and BD Patients

**Figure 2:**
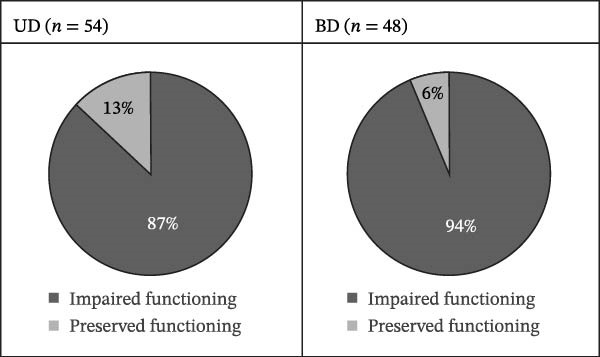
Global functioning impairment.

A comparative overview of global functioning measures for the UD and BD groups is reported in Table 2. According to FAST assessments, 87% (*n* = 47) of patients with UD and 93.7% (*n* = 45) of patients with BD showed clinically evident functional impairment, with scores below the normative threshold, as shown in Figure 2.

Comparison of mean FAST scores across each functional domain revealed significant differences between UD and BD patients in most domains. Specifically, patients with BD reported poorer global functioning than those with UD in all domains—autonomy (7.1 ± 3.4 vs. 5.8 ± 3.3, *p* = 0.031), cognitive functioning (10.4 ± 3.7 vs. 6.8 ± 3.1, *p*  < 0.001), occupational functioning (7.6 ± 4.1 vs. 6.7 ± 4.3, *p* = 0.034), financial issues (2.5 ± 2.1 vs. 1.4 ± 1.8, *p* = 0.003), and leisure time (4.7 ± 1.8 vs. 4.1 ± 1.9, *p* = 0.009)—except for interpersonal relationships (9.9 ± 4.4 vs. 8.4 ± 4.9, *p* = 0.281) (see Table 2). Sensitivity analyses confirmed that diagnostic category (UD vs. BD) remained a significant and independent predictor of global functional impairment (*F* = 5.732, *p* = 0.019, *η* ^2^_*p*_ = 0.074) after adjustment for illness recurrence. Notably, neither the total number of affective episodes (*p* = 0.103) nor the number of prior depressive episodes (*p* = 0.122) exerted a significant effect within the model, indicating that the greater level of disability observed in the BD group persists independently of patients’ recurrence history.

### 3.3. Correlation Between Global Functioning and Objective and Subjective Cognitive Performance

Pearson’s correlation coefficients were calculated to investigate the relationship between functional outcomes, as measured by the FAST, and cognitive functioning. Specifically, associations were examined between FAST scores and objective cognitive performance assessed with the SCIP, as well as between FAST scores and self‐reported cognitive difficulties evaluated using the PDQ‐D‐5.

**Figure 3:**
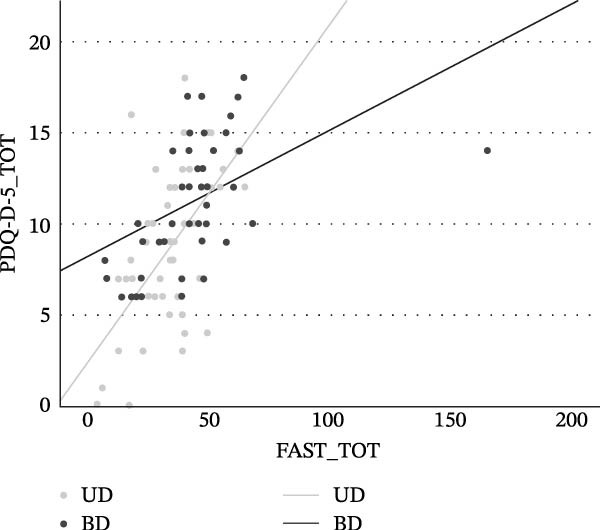
Correlation between global functioning and subjective cognitive performance in UD and BD patients.

In both the UD and BD groups, a statistically significant positive association was found between global functional impairment and subjectively perceived cognitive difficulties assessment (FAST vs. PDQ‐D‐5 in UD: *ρ* = 0.609, *p*  < 0.001; in BD: *ρ* = 0.475, *p*  < 0.001) (see Figure 3). Specifically, higher levels of functional impairment, as indicated by increasing FAST scores, were associated with greater self‐reported cognitive deficits, reflected by higher PDQ‐D‐5 scores.

**Figure 4:**
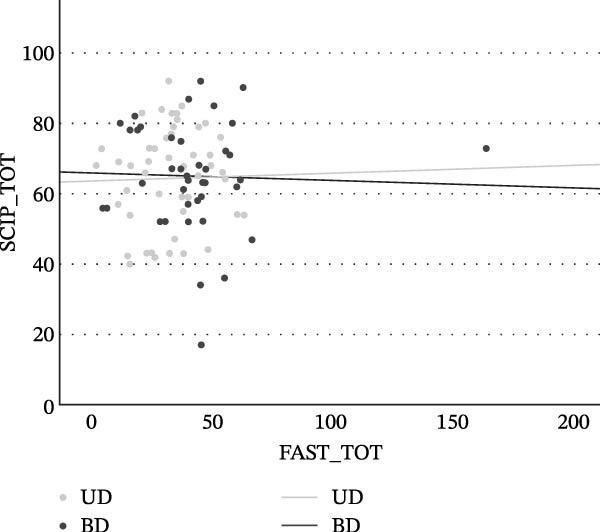
Correlation between global functioning and objective cognitive performance in UD and BD patients.

In contrast, no significant association was detected between functional performance and objectively measured cognitive abilities in either the UD or BD groups (FAST vs. SCIP in UD: *ρ* = 0.26, *p* = 0.851; in BD: *ρ* = −0.031, *p* = 0.834) (see Figure 4).

### 3.4. Correlation Between Global Functioning and Subjective Cognitive Subdomains

We subsequently examined potential associations between global functioning, as measured by the total FAST score, and the subjective cognitive subdomains of the PDQ‐D‐5, employing Pearson’s correlation analyses.

In the UD cohort, we observed significant positive correlations between global functioning and the cognitive subdomains of organization (*ρ* = 0.567, *p*  < 0.001), attention (*ρ* = 0.587, *p*  < 0.001), and orientation (*ρ* = 0.287, *p* = 0.035) (see Table 3). In contrast, within the BD group, significant positive associations were identified between functional outcomes and the subdomains of retrospective memory (*ρ* = 0.364, *p* = 0.011) and orientation (*ρ* = 0.371, *p* = 0.009) (see Table 4).

**Table 3:** Correlation between global functioning (assessed by FAST score) and subjective cognitive subdomains (assessed by PDQ‐5 subscores) in UD patients.

Item PDQ‐D‐5	Pearson correlation	*p*‐Value
Organization	0.567	**<0.001**
Attention	0.587	**<0.001**
Orientation	0.287	**0.035**
Retrospective memory	0.267	0.051
Prospective memory	0.163	0.238

*Note:* PDQ‐D‐5, perceived deficits questionnaire–depression–5‐items. The bold values indicate statistically significant differences (*p* < 0.05).

Abbreviations: FAST, functioning assessment short test; UD, unipolar depression.

**Table 4:** Correlation between global functioning (assessed by FAST score) and subjective cognitive subdomains (assessed by PDQ‐5 subscores) in BD patients.

Item PDQ‐D‐5	Pearson correlation	*p*‐Value
Organization	0.266	0.068
Attention	0.271	0.063
Orientation	0.371	**0.009**
Retrospective memory	0.364	**0.011**
Prospective memory	0.058	0.693

*Note:* PDQ‐D‐5, perceived deficits questionnaire–depression–5‐items. The bold values indicate statistically significant differences (*p* < 0.05).

Abbreviations: BD, bipolar depression; FAST, functioning assessment short test.

## 4. Discussion

Despite the increasing attention devoted in literature to global functioning in mood disorders, the specific contribution of neurocognitive impairments to functional outcomes in individuals with UD and BD remains incompletely characterized. By integrating measures of global functioning with assessments of both objective and subjective cognitive performance in individuals undergoing a MDE, this study provides original data on the relationship between cognitive dysfunction and functional impairment. Specifically, it delineates the strength and distribution of these associations across distinct cognitive subdomains.

This study was conducted in a real‐world cohort of individuals with UD and BD. The sociodemographic and clinical characteristics of the sample were consistent with those typically reported in patients with bipolar and depressive disorders and closely aligned with patterns described in the literature for individuals experiencing an MDE [35–37]. With respect to clinical variables, objective cognitive functioning was found to be impaired in both UD and BD, with no significant differences between diagnostic groups and a largely overlapping neurocognitive profile, in line with previous reports in the literature [21, 38–41].

In terms of global functioning, the findings of our study indicate that functional impairment is highly prevalent among individuals experiencing an MDE, with patients with BD exhibiting greater overall functional impairment than those with UD, both in total functioning and across most of functional subdomains. Comparisons of functional impairment during MDEs in individuals with unipolar versus bipolar disorders in the literature have yielded mixed and divergent findings. While studies by Godard et al. [42, 43] reported no significant differences in overall functional impairment between the two mood disorders, the majority of investigations are consistent with our findings, indicating a greater global functional burden in bipolar disorder, particularly within social, occupational, and household domains [12, 44, 45]. This discrepancy may be attributed to the use of different instruments to assess global functioning. Specifically, Godard and colleagues [42, 43] employed the Social adjustment scale (SAS), a self‐report measure widely used for general assessments of social functioning in psychiatric populations, but which is less sensitive to the specific functional impairments often observed in bipolar disorders compared to the FAST scale utilized in the present study. Our sensitivity analyses further elucidate that the functional disparity between UD and BD cannot be solely attributed to the greater number of episodes typically observed in bipolar populations. Rather, the findings suggest a distinct vulnerability within functional domains that is intrinsic to the bipolar depressive phase, thereby underscoring the necessity for diagnostic‐specific rehabilitative interventions.

Furthermore, we examined the relationship between subjective and objective cognitive measures and global functioning in the UD and BD groups. In our sample, we found a strong relationship between self‐reported cognitive performance and global functioning in both UD and BD patients, whereas no significant association emerged with objective cognitive measures. These findings are consistent with previous studies in the literature, which have reported that subjectively perceived cognitive impairment is strongly related to functional deficits in either diagnostic group [16, 18, 46]. Overall, subjectively assessed cognitive functioning may have some predictive value for functional outcomes in individuals with unipolar and bipolar disorders. Concerning objective cognitive evaluation, evidence in the literature remains heterogeneous and at times contradictory. Several studies are in line with our findings, indicating that objectively assessed cognitive deficits did not reliably predict nor substantially influence global functional capacity [18, 46–48]. Conversely, other investigations have reported an association between objective cognitive performance and overall functioning [49–51]. These discrepant findings, however, should be interpreted with caution in light of notable methodological limitations. In particular, many studies have employed heterogeneous samples, including individuals in different phases of illness, such as euthymic states and active depressive or (hypo)manic episodes [48, 50, 51]. Furthermore, substantial variability characterizes the assessment tools used to measure both objective cognition and global functioning, with functional outcomes frequently evaluated using general clinical indicators of functioning, as well as instruments such as the SAS or the global assessment of functioning (GAF), or even broader clinical indicators of functioning, which may lack adequate sensitivity for mood disorders, particularly bipolar disorder. Finally, our findings support the use of brief, disorder‐sensitive instruments that improve detection of relevant impairments in real‐world clinical settings. The FAST appears particularly sensitive to functional deficits associated with bipolar disorder compared with broader measures such as the GAF or SAS; however, it provides limited information on specific cognitive domains. As a brief measure of global functioning, it may miss subtle or domain‐specific deficits that can be captured by more detailed assessments such as the SCIP and PDQ‐D‐5. The SCIP allows rapid identification of objective cognitive impairments, whereas the PDQ‐D‐5 captures subjective cognitive difficulties, although it may be influenced by current mood state. Together, these instruments offer a pragmatic, multidimensional approach to assessing cognition and functional outcomes in patients with mood disorders [49–51].

When examining more specifically which areas of global functioning appear to be most compromised in relation to subjective cognitive functioning, our results appear particularly interesting and contribute to enriching the existing literature. Specifically, our findings highlight distinct patterns of functional impairment between UD and BD. In individuals with UD, impairments were more prominently observed in domains related to organization and attention, suggesting a specific vulnerability of executive‐related functioning. In contrast, in patients with BD, greater functional compromise emerged in memory‐related domains, pointing toward a differential cognitive–functional profile. These disorder‐specific patterns support the notion that subjective cognitive difficulties may translate into distinct functional impairments across diagnostic categories, underscoring the clinical relevance of tailoring assessment and intervention strategies accordingly.

The association between patients’ cognitive perceptions and real‐world functioning represents a clinically relevant area of investigation and warrants further examination through methodologically rigorous studies, particularly in view of its potential implications and the inconsistency of findings within the existing literature. If replicated, the present results may support previous evidence indicating that subjective cognitive difficulties constitute a meaningful dimension of mood disorders, with significant associations with functional outcomes and potential relevance for treatment planning and functional recovery. In this context, our findings underscore the importance of comprehensive clinical assessments integrating both objective and subjective measures of cognition and global functioning in the management of affective disorders, with the potential to support more personalized and precision‐oriented psychiatric care.

Several methodological considerations should be taken into account when interpreting the present findings. Pharmacological variables were not specifically examined in relation to depressive trajectories; nevertheless, no significant differences emerged between the BD and UD groups with respect to either the number or the classes of medications prescribed. The relatively limited sample size further constrains the robustness and generalizability of the results, underscoring the need for future investigations in larger and more representative cohorts. Moreover, the cross‐sectional nature of the study precluded the evaluation of longitudinal changes in cognitive and global functioning, which should be addressed in future research to better characterize the temporal dynamics of cognitive and functional outcomes in mood disorders. Finally, given the exploratory nature of the study and the correlations among cognitive and functional subdomains, we did not apply formal corrections for multiple comparisons (e.g., Bonferroni). Nominal *p*‐values were reported to balance the risk of Type I and Type II errors. Therefore, results should be interpreted as hypothesis‐generating, although the strongest associations (*p* <0 .001) remain highly robust.

Conversely, the current investigation exhibits several strengths. One key strength is the recruitment of a homogenous cohort of patients with unipolar and bipolar disorders specifically during the acute depressive phase, in contrast to prior studies that frequently incorporated heterogeneous samples spanning multiple illness states, including depressive, manic, and euthymic phases. Moreover, the study cohort was reflective of “real‐world” clinical populations, encompassing both inpatient and outpatient individuals with UD and BD, thereby enhancing the external validity and generalizability of the results. The present work further employed cognitive and functional assessment instruments characterized by high sensitivity, rapid administration, and clinical feasibility. Specifically, the SCIP and PDQ‐D‐5 were utilized for cognitive screening, while the FAST provided a measure of global functioning—tools that are readily implementable in routine clinical practice. By comparison, much of the extant literature used either less sensitive screening measures or comprehensive batteries that are time‐intensive and impractical for standard clinical settings.

## 5. Conclusions

In conclusion, this exploratory study provides a detailed characterization of the significant functional impairment in patients experiencing MDEs within both unipolar and bipolar disorders. Of particular interest is the observed correlation between subjective cognitive performance and functional measures across UD and BD patients, suggesting that perceived cognitive difficulties may exert a more substantial influence on daily functioning than objectively measurable cognitive deficits. Moreover, the distinct functional profiles related to cognitive performance identified in UD and BD patients underscore the importance of assessing specific domains of impairment to guide targeted interventions, aiming not only at symptomatic remission but also at functional recovery. Nevertheless, longitudinal investigations are warranted to elucidate the effects of pharmacological treatments and the trajectory of cognitive and global functioning over time, thereby clarifying the intricate relationship between cognitive impairments and functional outcomes in mood disorders.

## Author Contributions

Giorgia Porceddu and Gianluca Rosso designed the study. Giorgia Porceddu and Camilla Garrone collected the patients’ data. Giorgia Porceddu and Camilla Garrone managed literature search and analyzed the data. Giorgia Porceddu, Camilla Garrone, and Gianluca Rosso wrote the draft. Giuseppe Maina provided substantial comments and helped drafting the manuscript in its final form.

## Funding

This study was conducted without financial support from public, commercial, or not‐for‐profit funding bodies.

## Disclosure

All authors have read and approved the final version submitted and take public responsibility for all aspects of the work. All authors contributed to the study and are fully responsible for the content and conclusions of this article. No individuals or third‐party services not listed as authors were involved in the research or preparation of the manuscript without acknowledgment. All authors confirm that this manuscript has not been previously published elsewhere nor is it currently under consideration by any other journal, in whole or in part.

## Ethics Statement

All patients admitted to our inpatient and outpatient services provided written informed consent, approved by the local Ethics Committee, permitting the anonymous use of their sociodemographic and clinical data for research and educational purposes. All data were handled in accordance with applicable data protection regulations. The study was approved by our Ethical Committee (Protocol Number 71200206) and conducted in accordance with the principles of the Declaration of Helsinki, as revised at the 64th WMA General Assembly (Fortaleza, Brazil, October 2013).

## Conflicts of Interest

Gianluca Rosso is a speaker and consultant from Angelini, Janssen, Lundbeck, Otsuka, Rovi. Giuseppe Maina is a consultant and a speaker and/or has received research grants from Angelini, Boehringer Ingelheim, FB‐ Health, Janssen, Lundbeck, Otsuka, Innova Pharma. All other authors declare no conflicts of interest.

## Supporting Information

Additional supporting information can be found online in the Supporting Information section.

## Supporting information


**Supporting Information** The supporting information includes the STROBE checklist (Checklist STROBE_2.pdf) for cross‐sectional studies. The checklist provides a detailed account of adherence to STROBE reporting standards, with each item mapped to the corresponding sections of the manuscript. This ensures a transparent, complete, and methodologically rigorous reporting of the study design, data collection, statistical analyses, and interpretation of findings.

## Data Availability

The data supporting the findings of this study are available from the corresponding author upon reasonable request. The data are not publicly available due to confidentiality constraints related to information extracted from hospital clinical records.
