# Supplementary material for: Cognitive Symptoms and Overall Functioning During Major Depressive Episodes: Correlation Analysis of Patients With Unipolar and Bipolar Disorders
Source: Depress Anxiety. 2026 May 14;2026:7231049. doi: 10.1155/da/7231049 (PMC13175271; doi:10.1155/da/7231049)
Supplement: Supplementary file 1 — Supporting Information The supporting information includes the STROBE checklist (Checklist STROBE_2.pdf) for cross‐sectional studies. The checklist provides a detailed account of adherence to STROBE reporting standards, with each item mapped to the corresponding sections of the manuscript. This ensures a transparent, complete, and methodologically rigorous reporting of the study design, data collection, statistical analyses, and interpretation of findings. [file DA-2026-7231049-s001.pdf]

STROBE Statement—Checklist of items that should be included in reports of ***cross-sectional studies***

	Item No	Recommendation
**Title and abstract**	1	(*a*) cross-sectional observational study
		(*b*) **Aim**<br>This cross-sectional observational study was designed to characterize global functioning in patients with unipolar (UD) and bipolar depression (BD), focusing on the relationship between functional impairment and objectively assessed and subjectively perceived cognitive deficits, as well as to potential domain-specific cognitive–functional patterns across diagnostic groups.<br>**Methods**<br>Individuals experiencing a major depressive episode (MDE) in the context of major depressive or bipolar disorder were recruited. Global functioning was assessed with the Functional Assessment Short Test (FAST), objective cognition with the Screen for Cognitive Impairment in Psychiatry (SCIP), and subjective cognition with the Perceived Deficits Questionnaire–Depression–5-item (PDQ-D-5). Group differences were analyzed using χ^2 tests and ANCOVA, adjusting for illness duration and age at onset. Associations between cognitive measures and global functioning were examined using Pearson's correlations.<br>**Results**<br>A total of 102 patients were recruited: 54 with UD, 48 with BD. Clinically relevant functional impairment was observed in 87% of UD patients and 93.7% of BD patients. BD individuals showed greater global functional impairment than UD patients, with higher FAST total scores (44.5±23.1 vs. 33.5±14.6, p =.006) and worse functioning across most domains. Global functioning was strongly associated with subjectively perceived cognitive difficulties in both UD (ρ = 0.609, p <.001) and BD (ρ = 0.475, p <.001), whereas no significant associations were found with objective cognitive performance. Domain-specific analyses revealed different patterns of association, with attention and organization related to functioning in UD, and retrospective memory in BD.<br>**Conclusion**<br>MDEs in unipolar and bipolar disorders are associated with marked functional impairment. Perceived cognitive difficulties may impact daily functioning more than objectively assessed deficits, highlighting their clinical relevance. The distinct cognitive–functional profiles in UD and BD patients underscore the importance of domain-specific assessments to guide interventions targeting both symptom remission and functional recovery.
**Introduction**		
Background/rationale	2	Cognitive and global dysfunction has been increasingly recognized as a core feature of mood disorders, extending beyond acute symptomatology. Given the substantial burden of functional impairment during major depressive episodes and the emerging but inconsistent evidence on the role of cognitive dysfunction, further clarification of this relationship is warranted, particularly in light of the lack of integrated assessments of both subjective and objective cognition within clinically homogeneous samples.
Objectives	3	Aims:<br>1. Characterize overall functioning in patients with unipolar depression (UD) versus bipolar depression (BD);

		2. Examine the relationship between global functioning and objectively assessed vs subjectively perceived cognitive impairment;<br>3. Investigate whether specific cognitive domains impact on functioning.
**Methods**		
Study design	4	We conducted a cross-sectional observational study of adults experiencing a major depressive episode within the course of major depressive or bipolar disorders.
Setting	5	The study was conducted at the Psychiatric Unit of San Luigi Gonzaga University Hospital (University of Turin, Italy). Participants were recruited consecutively between December 2024 and August 2025. All assessments—including clinical interviews, self-report questionnaires, and clinician-administered cognitive and functional tests—were performed on site during this period, with no additional follow-up, as the study employed a cross-sectional design.
Participants	6	Eligibility criteria: participants were adults aged 18–65 years with at least 8 years of formal education, experiencing a major depressive episode within the course of major depressive disorder or bipolar disorder according to DSM-5-TR criteria. Exclusion criteria included a history of organic brain disorders, significant head trauma, intellectual disability, alcohol or substance use disorders, dementia, or other neurodegenerative diseases.<br>Sources and selection methods: participants were recruited consecutively among all patients with unipolar or bipolar depression referred to the Psychiatric Unit of San Luigi Gonzaga University Hospital (University of Turin, Italy) between December 2024 and August 2025.
Variables	7	Outcomes: objective cognitive functioning assessed with the Screen for Cognitive Impairment in Psychiatry (SCIP); subjective cognition assessed with the Perceived Deficits Questionnaire–Depression–5-item (PDQ-D-5), and global functioning assessed with the Functional Assessment Short Test (FAST).<br>Exposures/predictors: Diagnosis of major depressive disorder or bipolar disorder and severity of depressive symptoms according to the measures of the Hamilton Depression Rating Scale (HAM-D) and Beck Depression Inventory-II (BDI-II).<br>Potential confounders: Age, duration and age at onset of illness, and socio-demographic variables, for which the analyses were adjusted.<br>Effect modifiers: Type of depression (unipolar vs. bipolar).<br>Diagnostic criteria: Major depressive episode, major depressive disorder and bipolar disorder were defined according to DSM-5-TR criteria.
Data sources/ measurement	8*	Cognitive functioning was assessed objectively with the SCIP and subjectively with the PDQ-D-5; global functioning was measured using the clinician-rated FAST. Depressive symptoms were evaluated with HAM-D and BDI-II. All assessments were performed on site by trained clinicians using standardized procedures, and the same instruments were applied consistently across both unipolar and bipolar depression groups, ensuring comparability.
Bias	9	Bias was minimized through consecutive recruitment, exclusion of neurological or substance use disorders, standardized assessments, clinician-rated functional measures, and adjustment for age and illness duration.
Study size	10	The study size was determined by consecutive recruitment of all eligible patients referred during the study period, without a formal sample size calculation.
Quantitative variables	11	Continuous variables were analyzed as mean ± SD, and categorical variables as counts and percentages. Participants were grouped by diagnosis (unipolar vs. bipolar

		depression).
Statistical methods	12	(*a*) Statistical methods: Group differences were assessed using χ^2 tests for categorical variables and ANCOVA for continuous variables, controlling for age and illness duration.
		(*b*) Subgroups/interactions: Analyses were stratified by diagnosis (unipolar vs. bipolar depression); no formal interaction tests were performed.
		(*c*) Missing data: No missing data were observed.
		(*d*) Sampling strategy: Analyses were based on consecutive recruitment, with no additional sampling adjustments.
		(*e*) Sensitivity analyses: Not performed or applicable in this study.
**Results**		
Participants	13*	(a) Numbers at each stage: A total of 102 participants were included in the study, comprising 54 with unipolar depression (UD) and 48 with bipolar depression (BD). All participants who were eligible and consented were examined and analyzed.
		(b) Reasons for non-participation: no participants withdrew from the study.
		(c) Flow diagram: A flow diagram was not deemed necessary due to the absence of drop-outs or exclusions after enrolment.
Descriptive data	14*	(a) Participant characteristics: The study included 102 adults (54 UD, 48 BD). Groups were comparable in age, gender, years of education, smoking habits, and physical activity. BD patients had earlier illness onset and higher lifetime numbers of affective and depressive episodes than UD. Depressive symptom severity (HAM-D) and objective cognitive performance (SCIP) were similar between groups, whereas global functioning (FAST) was more impaired in BD. Exposures and potential confounders: The primary exposure was diagnosis (UD vs. BD), and potential confounders included age, duration and age at onset of illness, and socio-demographic factors.
		(b) Missing data: There were no missing data for any variables of interest.
Outcome data	15*	Functional impairment was observed in 87% of UD patients (n = 47) and 93.7% of BD patients (n = 45) according to FAST scores (≥15). Mean FAST scores were higher in BD than UD across most domains—autonomy, cognitive, occupational, financial, and leisure—while interpersonal relationships did not differ. Correlations showed FAST scores were positively associated with subjective cognitive difficulties (PDQ-D-5) but not with objective cognitive performance (SCIP), in both the UD and BD groups.
Main results	16	(*a*) Estimates: Group comparisons for continuous outcomes were performed using ANCOVA, controlling for age and illness duration as potential confounders; unadjusted mean values and standard deviations are reported (see Tables 2–4). 95% confidence intervals were not calculated.
		(*b*) Categorization of continuous variables: FAST scores were categorized as preserved functioning (<15) vs impaired (≥15), and SCIP scores were categorized as deteriorated vs non-deteriorated according to validated thresholds.
		(*c*) Absolute risk translation: Not applicable, as this was a cross-sectional observational study without follow-up or incidence data.
Other analyses	17	Correlation analyses were performed between global functioning (FAST) and both subjective (PDQ-D-5) and objective (SCIP) cognitive performance. Subdomain analyses examined associations between FAST and specific PDQ-D-5 cognitive dimensions, revealing significant correlations only with subjective cognitive

		measures in both UD and BD groups. No formal interaction or sensitivity analyses were conducted.
**Discussion**		
Key results	18	Functional impairment was common in both UD and BD, with greater deficits in BD. Subjective cognitive difficulties correlated with global functioning, while objective cognitive performance did not. Disorder-specific patterns emerged, with UD showing deficits in organization and attention and BD in memory domains, aligning with the study's objective of examining cognitive contributions to functional outcomes.
Limitations	19	Limitations include the cross-sectional design and modest sample size, which limit causal inference and generalizability. Unmeasured medication effects and reliance on subjective cognitive measures may introduce moderate bias, potentially inflating associations with functional impairment, but are unlikely to alter the main findings.
Interpretation	20	Overall, the study suggests that functional impairment is common in both UD and BD, with greater deficits in BD, and that subjective cognitive difficulties are more strongly associated with functioning than objective measures. These results should be interpreted cautiously but are broadly consistent with prior evidence. Longitudinal studies are needed to confirm these associations and clarify causal relationships.
Generalisability	21	The findings are likely generalizable to real-world adults with unipolar and bipolar depression in acute major depressive episodes, including both inpatients and outpatients. However, the modest sample size and recruitment from a single psychiatric unit may limit applicability to broader populations or other clinical settings.
**Other information**		
Funding	22	This study received no financial support from public, commercial, or not-for-profit organizations.

*Give information separately for exposed and unexposed groups.

Note: An Explanation and Elaboration article discusses each checklist item and gives methodological background and published examples of transparent reporting. The STROBE checklist is best used in conjunction with this article (freely available on the Web sites of PLoS Medicine at http://www.plosmedicine.org/, Annals of Internal Medicine at http://www.annals.org/, and Epidemiology at http://www.epidem.com/). Information on the STROBE Initiative is available at www.strobe-statement.org.
